# Transforaminal Endoscopic Versus Open Lumbar Foraminotomy for Lumbar Foraminal Stenosis: Comparative Two-Year Outcomes and Technical Considerations for Complete Endoscopic Decompression

**DOI:** 10.3390/jcm15176917

**Published:** 2026-09-07

**Authors:** Yong Ahn, Ik-Joon Choi, Sungsoo Bae, Dae-Jean Jo, Seong Son, Han-Byeol Park

**Affiliations:** 1 Department of Neurosurgery, Kyung Hee University Hospital at Gangdong, Kyung Hee University College of Medicine, Seoul 05278, Republic of Korea; sisyphusec@gmail.com (I.-J.C.); sungsoobae09@gmail.com (S.B.); apuzzo@hanmail.net (D.-J.J.); 2 Department of Neurosurgery, Gachon University Gil Medical Center, Incheon 21565, Republic of Korea; sonseong@gilhospital.com (S.S.); phbsgood@gilhospital.com (H.-B.P.)

**Keywords:** lumbar foraminal stenosis, transforaminal endoscopic lumbar foraminotomy, open lumbar foraminotomy, endoscopy, minimally invasive surgery

## Abstract

**Background/Objectives**: Transforaminal endoscopic lumbar foraminotomy (TELF) is increasingly used for lumbar foraminal stenosis, but direct comparisons with open lumbar foraminotomy remain limited. We compared 2-year clinical and perioperative outcomes between the procedures. **Methods**: We retrospectively reviewed consecutive patients who underwent single-level TELF or open lumbar foraminotomy between January 2020 and January 2024. The primary outcome was the change in leg-pain visual analog scale (VAS) score from baseline to 24 months. **Results**: Of 159 eligible patients, 147 completed 2-year follow-up (TELF, *n* = 72; open, *n* = 75). Improvement in leg-pain VAS score was 5.93 ± 1.60 points after TELF and 5.57 ± 1.44 points after open foraminotomy (mean difference, 0.36; 95% confidence interval, −0.14 to 0.85; *p* = 0.157). Two-year pain, disability, modified MacNab, complication, and reoperation outcomes did not differ significantly. TELF was associated with a shorter operative time (59.80 ± 15.37 vs. 81.20 ± 21.65 min), hospital stay (1.93 ± 0.68 vs. 6.84 ± 2.85 days), and return-to-work interval among patients who returned to work (3.91 ± 1.20 vs. 8.71 ± 2.45 weeks; all *p* < 0.001). **Conclusions**: TELF and open lumbar foraminotomy showed similar observed 2-year clinical outcomes, whereas the TELF treatment pathway was associated with faster perioperative recovery. These observational differences should not be interpreted as equivalence or as effects of surgical technique alone. Complete removal of the medial tip of the superior articular process is presented as a technical consideration for adequate foraminal decompression during TELF; its independent effect on outcomes was not evaluated.

## 1. Introduction

Lumbar foraminal stenosis can cause severe radiculopathy by compressing the exiting nerve root (ENR) through foraminal disc herniation, facet hypertrophy, ligamentum flavum thickening, or vertebral osteophytes [1]. Open lumbar foraminotomy using a microscopic paraspinal approach is an established motion-preserving treatment [2]. However, the restricted surgical corridor and need to preserve the facet joint can make adequate decompression technically challenging; nerve root manipulation or excessive facet resection may also contribute to postoperative dysesthesia, instability, or suboptimal outcomes [1,3,4].

Transforaminal endoscopic lumbar foraminotomy (TELF) provides direct foraminal decompression through a minimally invasive surgical corridor. Previous clinical studies and a systematic review have reported favorable outcomes after TELF [5,6,7]; however, the evidence is derived predominantly from single-arm studies, limiting conclusions regarding its outcomes relative to open surgery.

To our knowledge, no clinical cohort study has directly compared TELF with open lumbar foraminotomy. Therefore, we compared 2-year clinical and perioperative outcomes between these procedures in patients with symptomatic lumbar foraminal stenosis and described key technical considerations for consistent neural decompression during TELF.

## 2. Materials and Methods

### 2.1. Study Design and Outcomes

The primary outcome was the change in the visual analog scale (VAS) score for leg pain from baseline to 2 years. Perioperative recovery measures were secondary outcomes. A 1.6-point improvement was considered clinically important [8]. This observational cohort study was reported in accordance with the STROBE statement.

### 2.2. Patient Population

Clinical data routinely recorded during patient care were retrospectively reviewed for consecutive patients who underwent decompression for symptomatic lumbar foraminal stenosis between January 2020 and January 2024. The study was conducted in accordance with the Declaration of Helsinki and approved by the Institutional Review Board of Kyung Hee University Hospital at Gangdong (protocol code KHNMC 2026-04-033; 11 May 2026). The requirement for informed consent was waived by the Institutional Review Board because of the retrospective nature of the study.

The inclusion criteria were as follows: (1) single-level moderate-to-severe lumbar foraminal stenosis (Lee grade 2 or 3) on magnetic resonance imaging [9], with or without foraminal or extraforaminal disc herniation; (2) unilateral radiculopathy concordant with the radiographic findings; and (3) persistent symptoms despite at least 6 weeks of nonsurgical treatment, including medication, physical therapy, and/or selective nerve root block. The exclusion criteria were central canal stenosis, segmental instability, cauda equina syndrome, tumor, infection, previous surgery at the index level, and intracanal or paracentral disc herniation as the primary pathology.

The same clinical and radiographic eligibility criteria were applied to both groups. The surgical procedure was determined by the treating surgeon’s established practice. TELF was performed by two surgeons with established expertise in endoscopic spine surgery, whereas open lumbar foraminotomy was performed by two other surgeons experienced in microscopic lumbar decompression. Each surgeon exclusively performed the respective technique during the study period. All four surgeons had completed the initial learning phase for their respective procedures before the study period; no cases from the initial technique adoption were included. Nevertheless, surgeon experience was intrinsically linked to procedure and could not be modeled separately.

### 2.3. Surgical Techniques

#### 2.3.1. Transforaminal Endoscopic Lumbar Foraminotomy

TELF was performed under local anesthesia using an outside-in technique [5,10]. The procedure comprised three principal steps: (1) fluoroscopically guided transforaminal access to the stenotic foramen, (2) endoscopic bony decompression, and (3) removal of compressive soft tissue lesions. For premedication, midazolam (0.05 mg/kg) was administered intramuscularly, followed by intravenous fentanyl (0.8 μg/kg) immediately before the procedure. Under fluoroscopic guidance, the approach needle was advanced along a posterolateral transforaminal trajectory. The skin entry point and access angle were individualized according to the patient’s body habitus, operative level, and location of the foraminal pathology. An access angle of approximately 45° from the horizontal plane of the patient’s lower back was typically used. The needle was advanced toward the stenotic zone along the surface of the superior articular process (SAP) while avoiding the ENR. After fluoroscopic confirmation of needle placement, serial dilation was performed, and a bevel-ended working sheath was positioned at the outer aspect of the foramen. The sheath was not advanced directly into the intervertebral disc, and its bevel was directed away from the ENR. This outside-in position was intended to reduce the risk of neural injury and provide an appropriate working corridor for foraminal decompression.

**Figure 1:**
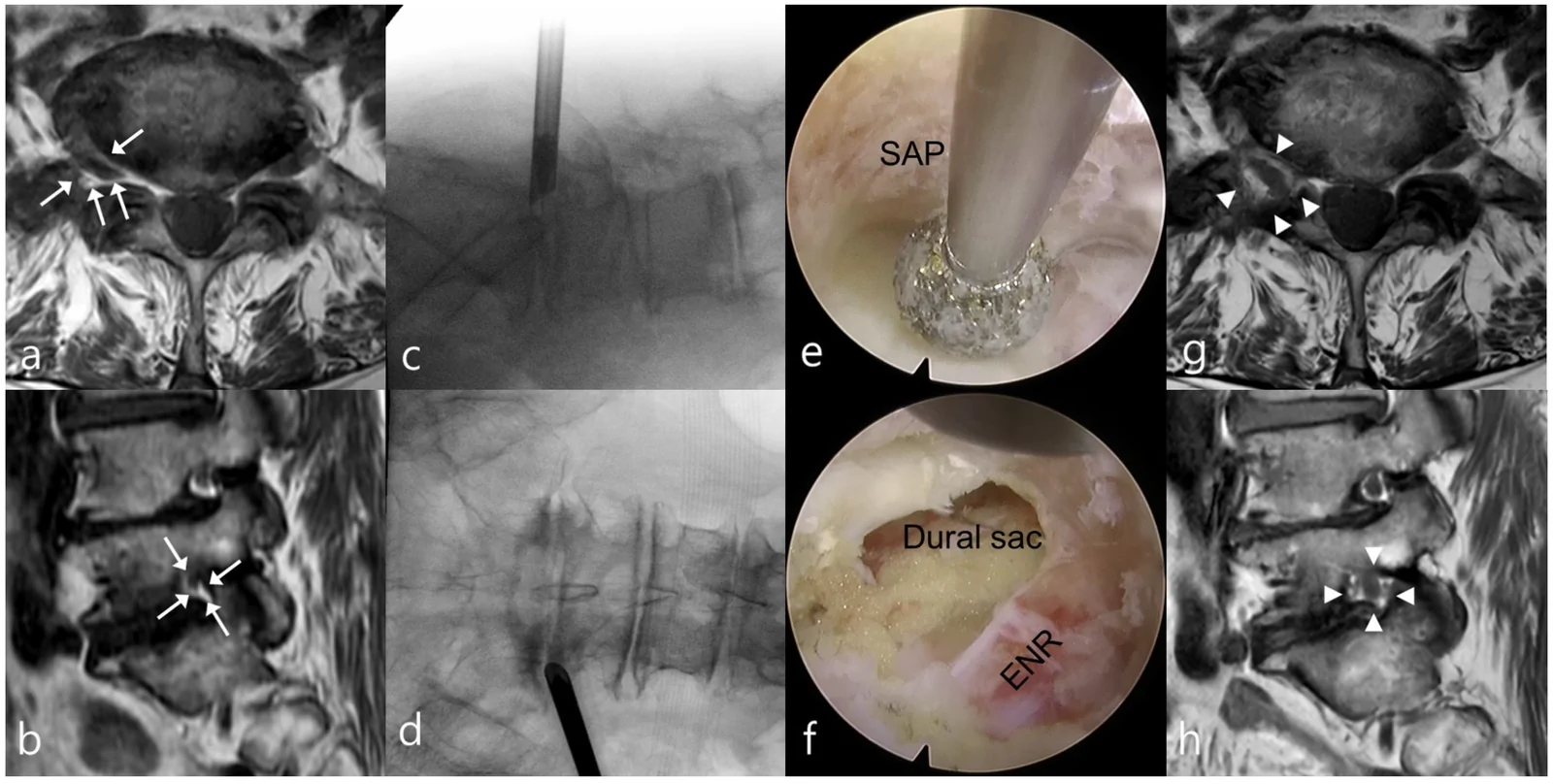
Representative case of TELF for lumbar foraminal stenosis. Preoperative axial and sagittal magnetic resonance imaging (MRI) images demonstrate severe foraminal stenosis caused by hypertrophy of the superior articular process (SAP) and ligamentum flavum (arrows) (**a**,**b**). Intraoperative fluoroscopic images show the position of the working sheath (**c**,**d**). Endoscopic views demonstrate SAP resection (**e**) and exposure of the exiting nerve root (ENR) and axillary epidural space after decompression (**f**). Postoperative axial and sagittal MRI images demonstrate adequate foraminal decompression (arrowheads) (**g**,**h**).

A working-channel endoscope with integrated viewing, working, and irrigation channels was subsequently introduced. After identifying the ENR, SAP, and disc space, the tip and lateral portions of the SAP were resected using an endoscopic burr and punches. Bony decompression was extended proximally along the course of the ENR until the ligamentum flavum and axillary epidural zone were exposed. The hypertrophied ligamentum flavum and other compressive soft tissues were removed using endoscopic punches, forceps, and radiofrequency probes. Selective discectomy was performed when foraminal or extraforaminal disc material contributed to ENR compression. Decompression was extended from the proximal axillary epidural zone to the lateral exit zone to release the ENR throughout the stenotic foramen. The procedural endpoint was adequate decompression with free mobilization and restored pulsation of the ENR on direct visualization (Figure 1).

#### 2.3.2. Open Lumbar Foraminotomy

Open lumbar foraminotomy was performed under general anesthesia using the standard paraspinal (Wiltse) approach [2]. After muscle-splitting exposure, a self-retaining retractor was placed. Under microscopic visualization, the lateral pars interarticularis and outer portion of the SAP were resected using a high-speed drill and Kerrison punch. The ligamentum flavum and other compressive tissues were removed to decompress the ENR from the proximal axillary epidural zone to the lateral exit zone. Selective discectomy was performed when foraminal or extraforaminal disc material contributed to ENR compression. Adequate decompression and free mobilization of the ENR were confirmed before layered wound closure with a subfascial drain.

### 2.4. Outcome Evaluation

Clinical outcomes were assessed preoperatively and at 6 weeks and 6, 12, and 24 months postoperatively. Leg- and back-pain VAS scores (0–10) [11] and ODI scores [12] were recorded. At 24 months, outcomes were graded using the modified MacNab criteria [13]. Clinical success was defined as an excellent or good result; a fair outcome indicated functional improvement requiring modification of daily activities, and a poor outcome indicated failure. Perioperative outcomes included operative time, hospital stay, return-to-work status, and return-to-work interval. Return to work was defined as resumption of preoperative or modified occupational duties after sick leave [14,15]. Return-to-work status was evaluated among patients employed preoperatively, whereas the interval was calculated among those who returned during follow-up. Complications included dural tear, hematoma, surgical-site infection, neurological deficit, and postoperative dysesthesia. Reoperation was defined as any additional index-level surgery within 24 months and was classified as a poor outcome. Follow-up outcomes were obtained at scheduled outpatient assessments or, when an in-person visit was not feasible, by a structured telephone interview. Postoperative analgesia and mobilization followed routine surgeon-specific care pathways rather than a unified study protocol; detailed medication regimens and exact mobilization timing were not consistently available for retrieval and were therefore not compared.

### 2.5. Statistical Analysis

Continuous variables are presented as mean ± standard deviation, and categorical variables as number (percentage) or number/denominator (percentage), as appropriate. Between-group comparisons were performed using the independent-samples *t*-test or Welch’s *t*-test for continuous variables and the chi-square test or Fisher’s exact test for categorical variables, as appropriate. Effect estimates with 95% confidence intervals were reported for the primary outcome and selected secondary outcomes to convey precision, particularly when event counts were small. No a priori sample-size calculation was performed because the sample comprised all eligible patients in this retrospective cohort.

The primary outcome was the change in leg-pain visual analog scale (VAS) score from baseline to 2 years. The mean change was compared between groups, and the between-group mean difference with its 95% confidence interval was calculated. Longitudinal changes in leg-pain VAS, back-pain VAS, and Oswestry Disability Index scores were evaluated using two-way repeated-measures analysis of variance, with time as the within-subject factor and group as the between-subject factor. Greenhouse–Geisser correction was applied when the assumption of sphericity was violated. Between-group comparisons at individual time points were performed using independent-samples *t*-tests and were considered exploratory. These time-point comparisons were not primary analyses, and no multiplicity adjustment was applied; their *p* values were interpreted descriptively.

The overall distribution of the modified MacNab categories was compared using the Fisher–Freeman–Halton exact test, and the proportion of excellent or good outcomes was compared using Fisher’s exact test. Time to return to work was analyzed among preoperatively employed patients who returned to work during follow-up. All tests were two-sided, and *p* < 0.05 was considered statistically significant. Statistical analyses were performed using IBM SPSS Statistics version 24.0 (IBM Corp., Armonk, NY, USA). In an additional time-to-event analysis, all patients employed preoperatively were included; return to work was treated as the event, and patients who did not return were censored at the 24-month follow-up. Kaplan–Meier curves were compared using the log-rank test. No multivariable adjustment was undertaken because the procedure was completely nested within the surgeon, precluding separation of procedure and surgeon effects.

### 2.6. Use of Generative Artificial Intelligence for Manuscript Preparation

During preparation of this manuscript, the authors used ChatGPT (OpenAI, GPT-5.6 Sol) solely to assist with English-language editing, clarity, and phrasing. The tool was not used to generate or analyze study data, perform statistical analyses, select results, or make clinical or scientific decisions. All AI-assisted text was critically reviewed and edited by the authors, who take full responsibility for the content of the publication.

## 3. Results

### 3.1. Patient Enrollment and Baseline Characteristics

During the study period, 159 patients met the inclusion criteria. Two-year follow-up was completed by 72 of 76 patients (94.7%) treated with TELF and 75 of 83 patients (90.4%) treated with open lumbar foraminotomy. Therefore, 147 patients were included in the final analysis. Baseline characteristics are summarized in Table 1. There were no significant between-group differences in sex distribution, age, body mass index, operative level distribution, or preoperative clinical scores. The 12 patients who did not complete 2-year follow-up were excluded from the complete-case analysis, and no outcome values were imputed. Given the small number of lost patients and the absence of additional retrievable baseline variables in the analytic dataset, a formal comparison between completers and noncompleters was not performed; attrition bias therefore remains possible.

### 3.2. Clinical Outcomes

**Figure 2:**
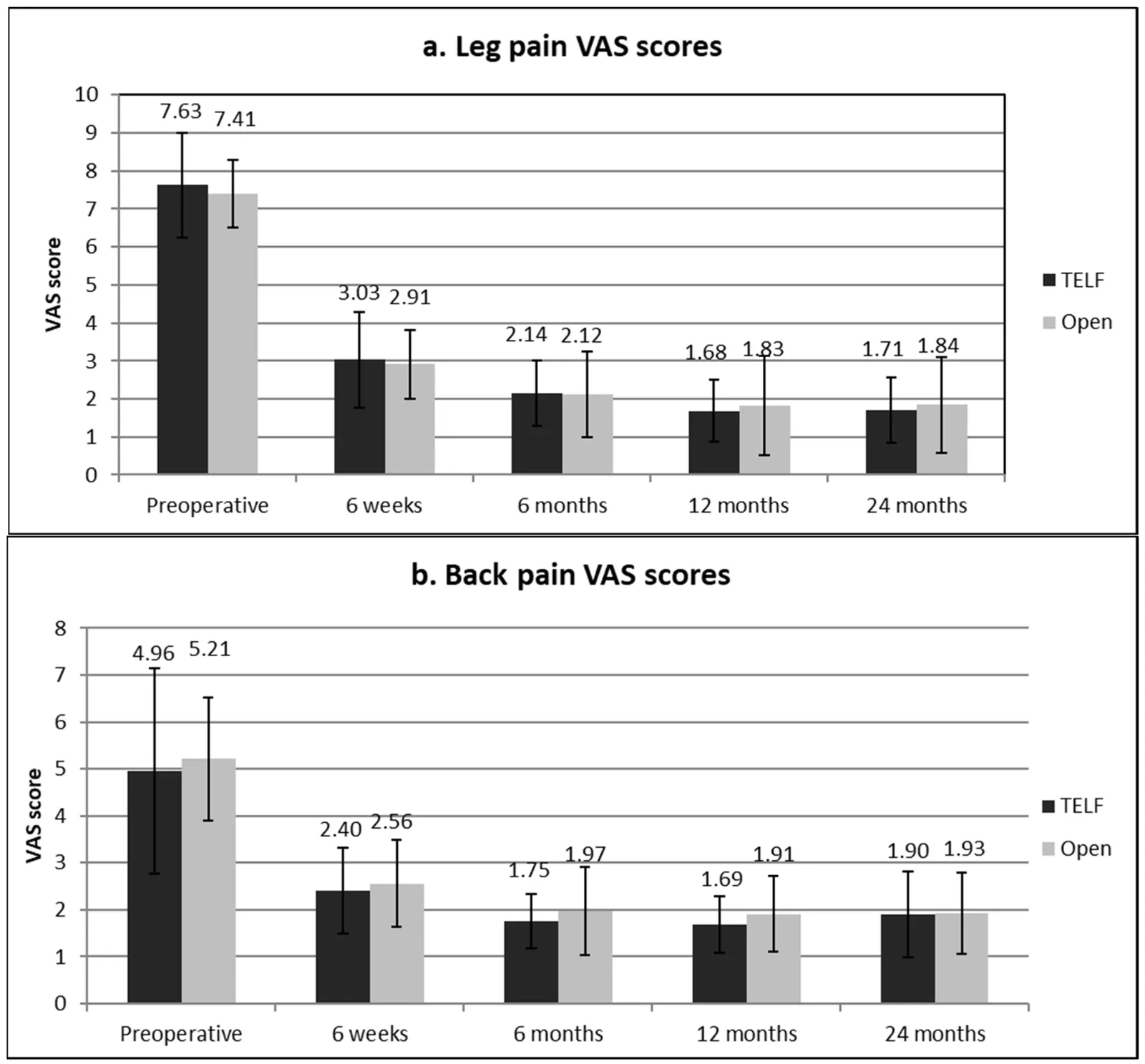

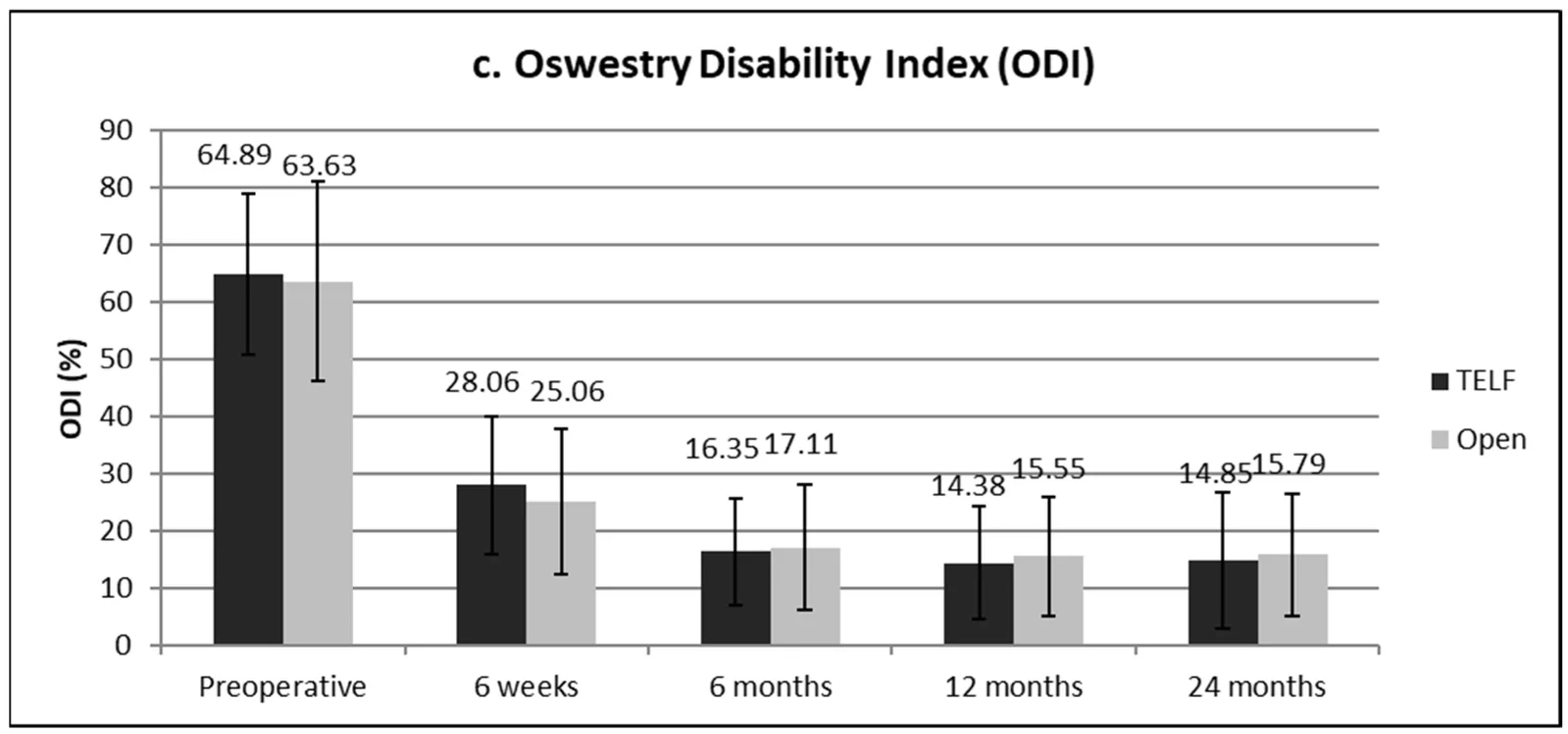
Serial changes in leg-pain visual analog scale (VAS) scores (**a**), back-pain VAS scores (**b**), and Oswestry Disability Index (ODI) scores (**c**) during the 24-month follow-up. Data are presented as mean ± standard deviation.

Leg-pain VAS, back-pain VAS, and ODI scores improved significantly over time (time effect, all *p* < 0.001), without significant group-by-time interactions (*p* = 0.283, *p* = 0.686, and *p* = 0.231, respectively) (Figure 2 and Table 2). The primary outcome, improvement in the leg-pain VAS score from baseline to 24 months, was 5.93 ± 1.60 points in the TELF group and 5.57 ± 1.44 points in the open group. The between-group mean difference was 0.36 points (95% confidence interval, −0.14 to 0.85; *p* = 0.157). At 24 months, leg-pain VAS scores were 1.71 ± 0.86 in the TELF group and 1.84 ± 1.25 in the open group (*p* = 0.458); back-pain VAS scores were 1.90 ± 0.92 and 1.93 ± 0.86, respectively (*p* = 0.836); and ODI scores were 14.85 ± 11.89 and 15.79 ± 10.65, respectively (*p* = 0.617). No significant between-group differences in leg-pain VAS, back-pain VAS, or ODI scores were observed at any assessment point. Complete leg-pain VAS, back-pain VAS, and ODI observations were available for all 147 analyzed patients at each prespecified assessment; therefore, the repeated-measures analysis used complete longitudinal data.

At 24 months, modified MacNab outcomes were excellent, good, fair, and poor in 13 (18.1%), 49 (68.1%), 7 (9.7%), and 3 patients (4.2%), respectively, in the TELF group and 12 (16.0%), 50 (66.7%), 9 (12.0%), and 4 patients (5.3%), respectively, in the open group. The distributions did not differ significantly between groups (*p* = 0.970). Clinical success was achieved by 62 patients in each group (TELF, 86.1%; open, 82.7%; Fisher’s exact test, *p* = 0.652) (Table 3).

### 3.3. Perioperative Outcomes and Return to Work

**Figure 3:**
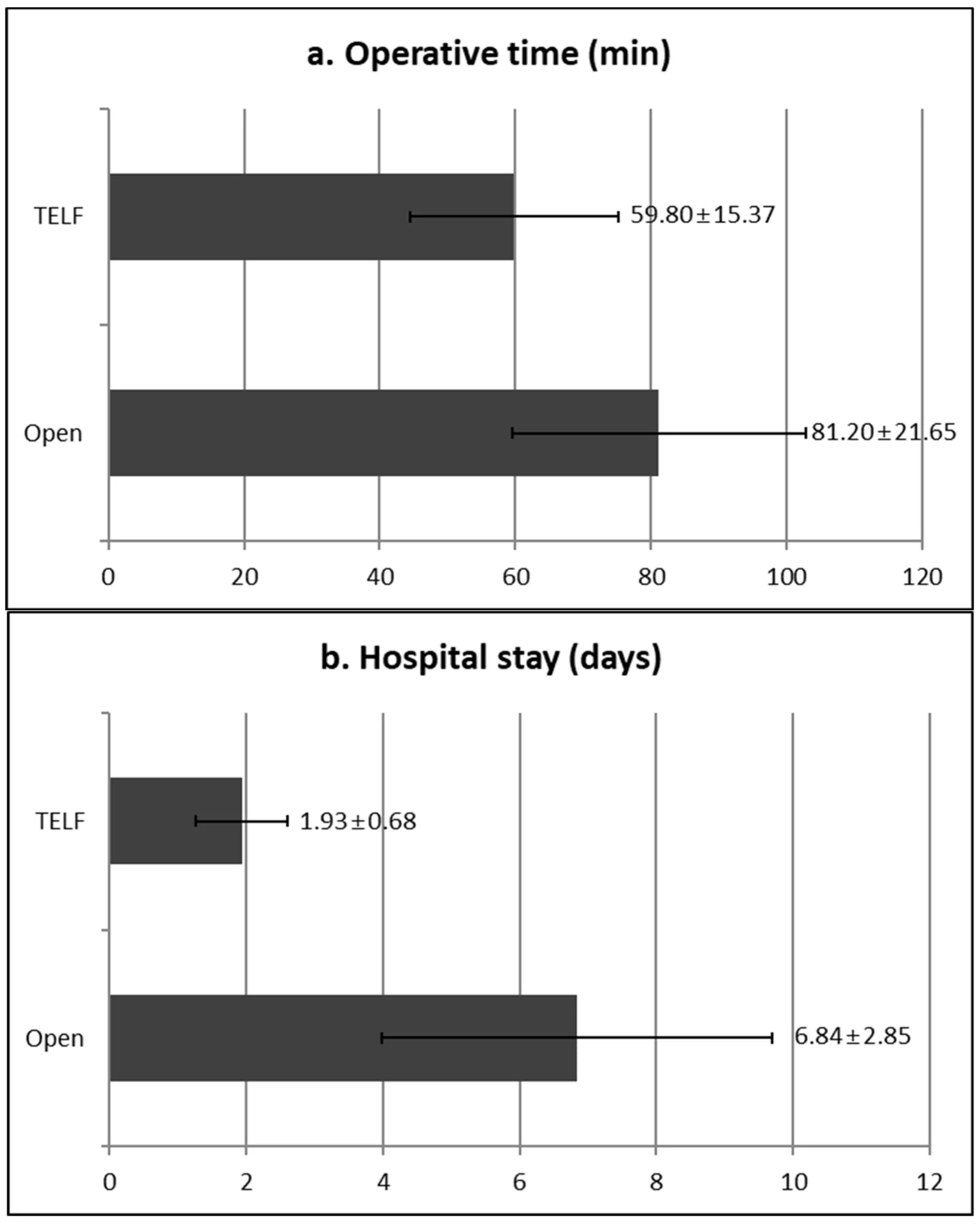

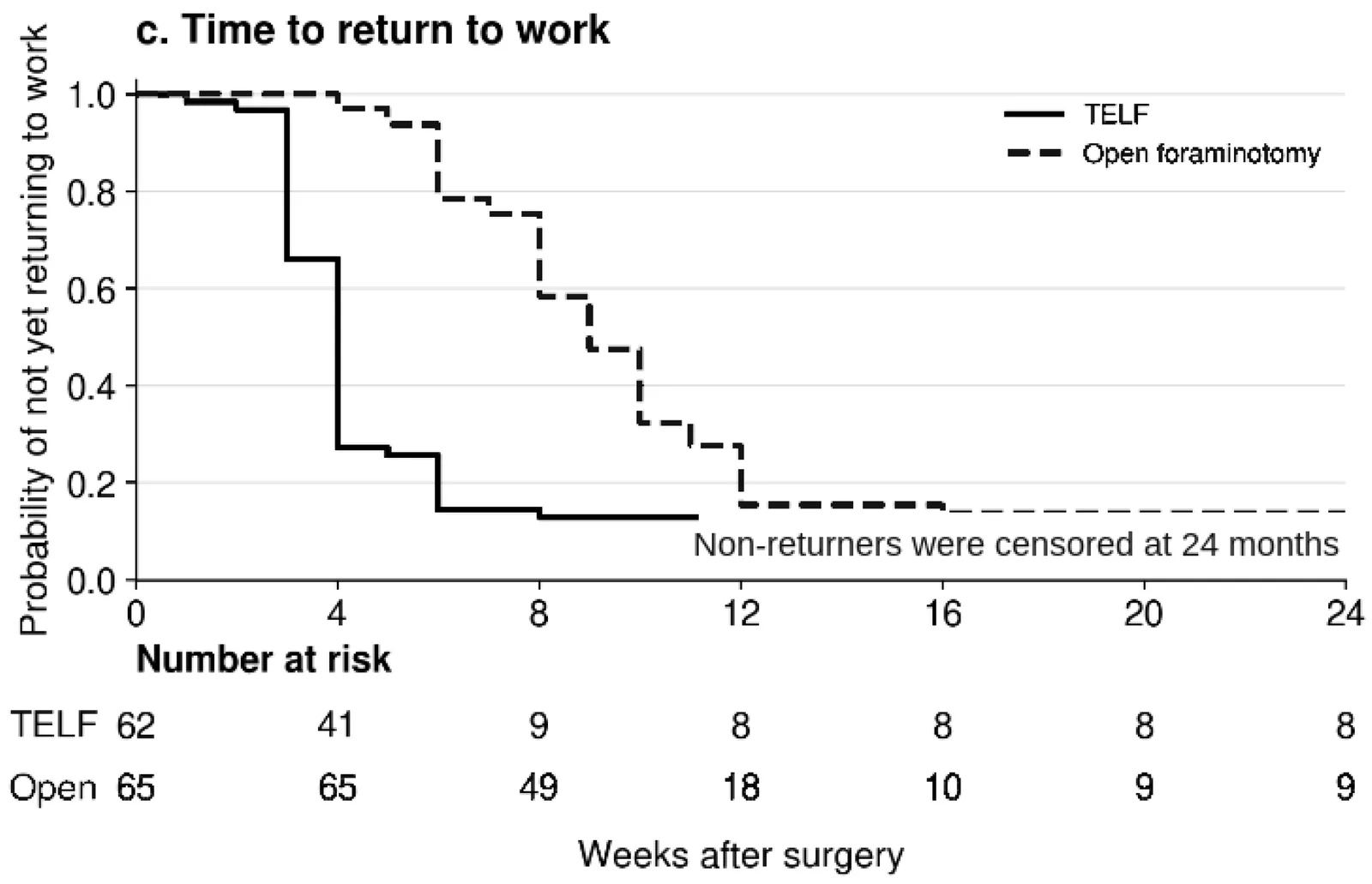
Comparison of perioperative outcomes between the TELF and open lumbar foraminotomy groups. Operative time (**a**) and length of hospital stay (**b**) were shorter in the TELF group. Kaplan–Meier curves showing time to return to work among all patients employed preoperatively (**c**). Return to work was treated as the event, and patients who did not return were censored at 24 months. The median time to return to work was 4 weeks after TELF and 9 weeks after open foraminotomy (log-rank *p* < 0.001). TELF, transforaminal endoscopic lumbar foraminotomy.

TELF was associated with a shorter operative time (59.80 ± 15.37 vs. 81.20 ± 21.65 min; *p* < 0.001) and hospital stay (1.93 ± 0.68 vs. 6.84 ± 2.85 days; *p* < 0.001) (Table 3 and Figure 3). Of 62 patients in the TELF group and 65 in the open group who were employed preoperatively, 54 (87.1%) and 55 (84.6%), respectively, returned to work during follow-up (*p* = 0.801). Among patients who returned to work, the time to return was shorter in the TELF group (3.91 ± 1.20 vs. 8.71 ± 2.45 weeks; *p* < 0.001). In the time-to-event analysis, including all 127 patients employed preoperatively, the median time to return to work was 4 weeks after TELF and 9 weeks after open foraminotomy (log-rank *p* < 0.001); the 17 patients who did not return were censored at 24 months.

### 3.4. Complications and Reoperation

No significant between-group differences were observed in the rates of individual complications. Incidental dural tears occurred in two patients in each group (*p* = 1.000) without subsequent cerebrospinal fluid-related complications. Postoperative dysesthesia occurred in six patients in the TELF group and five in the open group (8.3% vs. 6.7%; *p* = 0.762); all cases resolved with conservative treatment. Superficial surgical-site infection occurred in two patients in the open group, and postoperative hematoma occurred in one patient in the open group. Index-level reoperation was performed in three patients in each group (4.2% vs. 4.0%; *p* = 1.000). In the TELF group, two patients underwent revision TELF and one underwent fusion, whereas all three patients who underwent reoperation in the open group underwent fusion. The TELF-versus-open risk differences were 1.7 percentage points for postoperative dysesthesia (95% confidence interval, −7.5 to 11.1), 0.1 percentage points for dural tear (−6.7 to 7.2), and 0.2 percentage points for index-level reoperation (−7.5 to 8.0), underscoring the imprecision arising from the small number of events.

## 4. Discussion

### 4.1. Interpretation of Clinical Outcomes

Leg-pain VAS, back-pain VAS, and ODI scores improved significantly over time in both groups, with no significant group-by-time interactions, indicating that the trajectories of postoperative recovery did not differ significantly between the two procedures. The mean improvements in the leg pain VAS score exceeded the reported 1.6-point minimal clinically important difference after lumbar spine surgery in both groups [8]. Interpretation of these improvements should account for the threshold used to define clinically important change and clinical factors, such as symptom duration, that may influence postoperative recovery [16,17]. However, the between-group difference in improvement was not significant (mean difference, 0.36; 95% confidence interval, −0.14 to 0.85). No significant between-group differences were observed in VAS or ODI scores, modified MacNab outcome distributions, or clinical success rates at 2 years. Collectively, these findings suggest broadly similar observed clinical outcomes after TELF and open lumbar foraminotomy. The 1.6-point threshold was used only to contextualize the magnitude of within-group improvement; it was not a prespecified margin for equivalence or non-inferiority. Although the 95% confidence interval for the between-group mean difference was narrower than this threshold, the observational design and residual confounding preclude a formal claim of equivalence.

Complication and reoperation rates were low and did not differ significantly between groups. Postoperative dysesthesia occurred after both procedures, resolved with conservative treatment, and may reflect manipulation of the ENR or dorsal root ganglion rather than an endoscopy-specific complication [4]. Incidental dural tears were infrequent and were not followed by cerebrospinal fluid-related complications. Superficial infection occurred only after open foraminotomy; however, a few events precluded meaningful comparisons. The numerically higher dysesthesia rate after TELF was not statistically distinguishable from that after open surgery, and the wide confidence interval was compatible with either benefit or harm. Possible contributors include ENR or dorsal root ganglion manipulation and unmeasured surgeon-related factors; the present data cannot distinguish these mechanisms or establish a learning-curve effect.

### 4.2. Perioperative Advantages of TELF

The principal between-group differences involved perioperative recovery. TELF was associated with an approximately 21-min shorter operative time, 4.9-day shorter hospital stay, and, among patients who returned to work, 4.8-week shorter return-to-work interval. These findings are consistent with the tissue-preserving transforaminal approach, which minimizes paraspinal muscle disruption and provides targeted access to foraminal pathology [18,19,20,21,22,23]. Favorable outcomes and short hospital stays have also been reported after full-endoscopic foraminal decompression [24]. The analysis including all preoperatively employed patients likewise showed an earlier return-to-work distribution after TELF; however, this finding remains subject to the same treatment-pathway and occupational confounding.

However, these differences cannot be solely attributed to the surgical approach. TELF and open foraminotomy differed in anesthesia, drain use, postoperative protocols, and surgeon-specific practice, all of which may have influenced operative time and discharge. Return to work is also influenced by occupational demands, employer policies, and socioeconomic factors. These perioperative differences should therefore be interpreted as outcomes of the respective treatment pathways rather than isolated effects of endoscopy or evidence of cost-effectiveness. Thus, operative time, hospital stay, and return to work are composite perioperative outcomes influenced by anesthesia, drain use, postoperative care, occupational demands, and patient-specific factors, and should not be interpreted as purely technique-driven advantages.

### 4.3. Landmark-Guided Strategy for Complete Foraminal Decompression

Successful minimally invasive lumbar foraminotomy, including TELF, requires decompression of the ENR from the proximal axillary region to the distal exit zone rather than enlargement of the lateral foramen alone. A key technical consideration is complete removal of the hypertrophied SAP tip to its medial margin. In our experience, an unrecognized medial SAP remnant may contribute to incomplete foraminotomy by maintaining proximal ENR compression despite an apparently widened outer foramen. Importantly, simple exposure of the ENR may lead surgeons to overlook this remnant. This step was used as part of the intended TELF decompression strategy throughout the study period; however, postoperative CT- or MRI-based quantification of SAP resection was not performed systematically.

**Figure 4:**
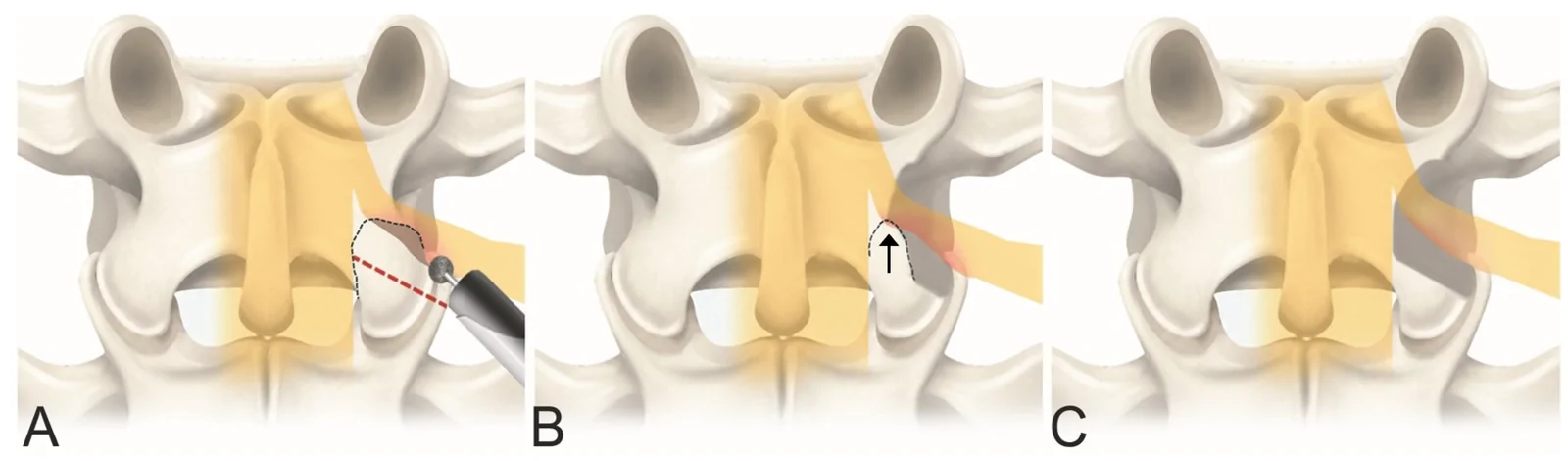
Key technical step for complete foraminal decompression during TELF, shown in the dorsal view. Endoscopic foraminal decompression is performed by removing the superior articular process (SAP) tip within the area outlined by the dotted line (**A**). Exposure of the exiting nerve root (ENR) alone, with a residual medial SAP tip (arrow), may result in incomplete decompression (**B**). Complete removal of the medial SAP tip exposes both the ENR and its axillary epidural space, allowing thorough foraminal decompression (**C**).

The facet joint cleft between the SAP and inferior articular process (IAP) served as an anatomical landmark for complete bony unroofing. After the synovial cleft was identified, the SAP tip was followed medially and removed completely, with the decompression margin extending towards the IAP and isthmus (Figure 4). The ligamentum flavum and other compressive tissues were removed until the proximal axillary epidural space was visualized. Decompression was continued distally along the ENR to the lateral exit zone (Figure 4). Complete release and free mobilization of the ENR, together with restored neural pulsation, served as procedural endpoints. This landmark-guided sequence may provide an anatomical framework for comprehensive decompression and help prevent residual proximal compression; however, its independent contribution to long-term clinical outcomes was not evaluated. Accordingly, the medial SAP tip is presented as an experience-based anatomical landmark and technical consideration, not as a radiographically validated mediator of superior clinical outcomes in this cohort.

### 4.4. Limitations

This study had several limitations. Its retrospective, non-randomized design made it susceptible to selection bias and residual confounding. Although both groups were treated using the same clinical indications and required Lee grade 2 or 3 foraminal stenosis, patient-level Lee-grade distributions, foraminal dimensions, symptom duration, concomitant disc herniation, comorbidity burden, and occupational demand were not available for comparative adjustment. Consequently, preferential selection of TELF for more favorable anatomy cannot be excluded. Treatment was determined by the surgeon’s established practice, and because surgeons were completely nested within procedure, surgeon- and technique-related effects could not be separated statistically. Although all surgeons were beyond their initial learning phase, this design does not exclude differences in proficiency or perioperative practice. Postoperative analgesia, mobilization, anesthesia, and drain use were not standardized across groups; perioperative outcomes therefore reflect the overall treatment pathways rather than surgical technique alone. Postoperative foraminal enlargement, completeness of medial SAP-tip removal, facet preservation, and segmental stability were not evaluated systematically. Return to work remained susceptible to occupational and socioeconomic confounding, despite the inclusion of non-returners as censored observations in the time-to-event analysis. Twelve of 159 eligible patients were excluded after loss to 2-year follow-up, no imputation was performed, and attrition bias cannot be excluded. No a priori sample-size calculation was performed, and the small number of adverse events resulted in wide confidence intervals. The findings may not be generalizable to surgeons in the TELF learning phase, and the 2-year follow-up may not capture late recurrence or instability. Future prospective multicenter studies should compare the procedures using standardized perioperative pathways, surgeon-balanced treatment allocation, quantitative foraminal imaging, and prespecified patient-centered recovery outcomes to better distinguish procedure-related effects from treatment-pathway and surgeon-related effects. Finally, the study was not designed as an equivalence or non-inferiority trial; the absence of statistically significant between-group differences should not be interpreted as proof of equivalence.

## 5. Conclusions

TELF and open lumbar foraminotomy showed no statistically significant differences in observed 2-year clinical outcomes in this retrospective cohort; this finding does not establish equivalence. The TELF treatment pathway was associated with a shorter operative time, hospital stay, and return to work, but these outcomes were inseparable from the effects of anesthesia, postoperative care, surgeon, and patient factors. Complete removal of the medial SAP tip is presented as a technical consideration for adequate foraminal decompression during TELF, although its independent effect on outcomes was not evaluated.

## Figures and Tables

**Table 1:** Baseline characteristics.

Characteristic	TELF (*n* = 72)	Open (*n* = 75)	*p* Value
Sex, male:female	29:43	36:39	0.407
Age, years	63.9 ± 10.5	62.7 ± 10.9	0.498
BMI, kg/m^2^	23.7 ± 2.7	23.8 ± 2.8	0.826
Operative level, *n* (%)			0.733
L1–2	0 (0.0)	0 (0.0)	
L2–3	1 (1.4)	2 (2.7)	
L3–4	5 (6.9)	4 (5.3)	
L4–5	28 (38.9)	24 (32.0)	
L5–S1	38 (52.8)	45 (60.0)	
Preoperative back-pain VAS score	4.96 ± 2.19	5.21 ± 1.31	0.396
Preoperative leg-pain VAS score	7.63 ± 1.38	7.41 ± 0.89	0.242
Preoperative ODI score	64.89 ± 14.02	63.63 ± 17.52	0.631

Data are presented as mean ± standard deviation or *n* (%). TELF, transforaminal endoscopic lumbar foraminotomy; BMI, body mass index; VAS, visual analog scale; ODI, Oswestry Disability Index.

**Table 2:** Longitudinal analysis of clinical outcomes.

Outcome	Time Effect, *p* Value	Group Effect, *p* Value	Group-by-Time Interaction, *p* Value
Leg-pain VAS	<0.001	0.890	0.283
Back-pain VAS	<0.001	0.144	0.686
ODI	<0.001	0.852	0.231

*p* values were obtained using two-way repeated-measures analysis of variance with Greenhouse–Geisser correction when sphericity was violated. VAS, visual analog scale; ODI, Oswestry Disability Index.

**Table 3:** Perioperative data and overall clinical outcomes.

Outcome	TELF (*n* = 72)	Open (*n* = 75)	*p* Value
Perioperative outcomes			
Operative time, min	59.80 ± 15.37	81.20 ± 21.65	<0.001
Hospital stay, days	1.93 ± 0.68	6.84 ± 2.85	<0.001
Employed preoperatively, *n*/*N* (%)	62/72 (86.1)	65/75 (86.7)	1.000
Returned to work, *n*/*N* (%) ^a^	54/62 (87.1)	55/65 (84.6)	0.801
Mean time to return to work, weeks ^b^	3.91 ± 1.20	8.71 ± 2.45	<0.001
Median time to return to work, weeks ^c^	4	9	<0.001
Complications, *n* (%)			
Superficial surgical-site infection	0 (0.0)	2 (2.7)	0.497
Postoperative hematoma	0 (0.0)	1 (1.3)	1.000
Dural tear	2 (2.8)	2 (2.7)	1.000
Postoperative dysesthesia	6 (8.3)	5 (6.7)	0.762
Index-level reoperation, *n* (%)	3 (4.2)	3 (4.0)	1.000
Modified MacNab outcome, *n* (%)			0.970
Excellent	13 (18.1)	12 (16.0)	
Good	49 (68.1)	50 (66.7)	
Fair	7 (9.7)	9 (12.0)	
Poor	3 (4.2)	4 (5.3)	
Excellent or good outcome	62 (86.1)	62 (82.7)	0.652

Data are presented as *n* (%), *n*/*N* (%), or mean ± standard deviation. ^a^ Calculated among patients employed preoperatively. ^b^ Calculated among preoperatively employed patients who returned to work during follow-up. ^c^ Kaplan–Meier median among all patients employed preoperatively; patients who did not return were censored at 24 months. The *p* value was obtained using the log-rank test. TELF, transforaminal endoscopic lumbar foraminotomy.

## Data Availability

The data presented in this study are not publicly available because they contain information that could compromise participant privacy. De-identified data may be available from the corresponding author upon reasonable request, subject to institutional and ethical approval.

## Abbreviations

BMI	body mass index
ENR	exiting nerve root
IAP	inferior articular process
MRI	magnetic resonance imaging
ODI	Oswestry Disability Index
SAP	superior articular process
TELF	transforaminal endoscopic lumbar foraminotomy
VAS	visual analog scale
